# Practical Notes on Diagnosis and Treatment

**Published:** 1912-02-03

**Authors:** 


### Practical Notes on Diagnosis and Treatment.

### Asthma.

Nocturnal attacks of asthma may often be prevented by the administration of ʒss to ʒj of ethereal tincture of lobelia at bed-time.

### Cod-liver Oil.

When the administration of cod-liver oil causes nausea and vomiting, it will sometimes agree if a dose of sodium bicarbonate and infusion of gentian is given before the meal and the oil immediately after the meal.

### Chronic Arthritis.

Considerable relief from the pain and stiffness of chronic arthritis may be obtained by the use of salt packs. An old piece of flannel is wrung out of brine and wrapped round the joint, and over this is placed a layer or two of dry flannel and the application worn all night.

### Pediculus Pubis.

One of the most effective and cleanly applications is a solution of perchloride of mercury. The following is a suitable formula: Perchloride of mercury, 8 grains; rectified spirit, ℥iss; solution of cochineal, ʒj; water to ℥iv. The cochineal, by colouring the solution, lessens the risk of accidental poisoning.

### Bronchitis in Children.

Oil of eucalyptus is distinctly helpful as an inhalation in the bronchitis of children. It may be made into a liniment with olive oil (1 in 8) and rubbed on the chest two or three times a day; or mixed with water (1 in 6) and the tent surrounding the cot moistened with the fluid. In either case the inhaled air carries with it some of the oil and lessens irritation and cough.

### Acute Tonsillitis.

Of the various drugs advised in follicular tonsillitis none acts so satisfactorily as antipyrine. The backache and headache and high temperature speedily subside under a few 10-grain doses of this drug. Locally the best gargle is cocaine hydrochloride, 8 grains; glycerine, ʒiv; carbolic acid, ʒj; rose water to ℥xij. The carbolic acid acts as a local anæsthetic, tends to paralyse the palatal muscles, and prevents the painful attempts at swallowing saliva and mucus.—*Sir William Whitla.*

### The Dosage of Bismuth Salts.

Subnitrate and other salts of bismuth are often given unsuccessfully because too small a dose is employed. Thirty grains, or even more, given in a cachet before food will often prevent gastric pain where 10 or 15 grains have proved useless. If it is desired to order the bismuth salt in a mixture it may be prescribed with compound infusion of orange, the mixture being shaken immediately before each administration. This is really more satisfactory than the use of tragacanth or other suspending agent.

